# Constructing the boundary between potent and ineffective siRNAs by MG-algorithm with C-features

**DOI:** 10.1186/s12859-022-04867-9

**Published:** 2022-08-13

**Authors:** Xingang Jia, Qiuhong Han, Zuhong Lu

**Affiliations:** 1 School of Mathematics, Southeast University, Nanjing, 210096 People’s Republic of China; 2 Department of Mathematics, Nanjing Forestry University, Nanjing, 210037 People’s Republic of China; 3 State Key Laboratory of Bioelectronics, School of Biological Science and Medical Engineering, Southeast University, Nanjing, 210096 People’s Republic of China

**Keywords:** MG-algorithm, *Icc*-cluster, *C*-feature, *I*-iteration

## Abstract

**Background:**

In siRNA based antiviral therapeutics, selection of potent siRNAs is an indispensable step, but these commonly used features are unable to construct the boundary between potent and ineffective siRNAs.

**Results:**

Here, we select potent siRNAs by removing ineffective ones, where these conditions for removals are constructed by *C*-features of siRNAs, *C*-features are generated by *MG*-algorithm, *Icc*-cluster and the different combinations of some commonly used features, *MG*-algorithm and *Icc*-cluster are two different algorithms to search the nearest siRNA neighbors. For the ineffective siRNAs in test data, they are removed from test data by *I*-iteration, where *I*-iteration continually updates training data by adding these successively removed siRNAs. Furthermore, the efficacy of siRNAs of test data is predicted by their nearest neighbors of training data.

**Conclusions:**

By siRNAs of Hencken dataset, results show that our algorithm removes almost ineffective siRNAs from test data, gives the clear boundary between potent and ineffective siRNAs, and accurately predicts the efficacy of siRNAs also. We suggest that our algorithm can provide new insights for selecting the potent siRNAs.

**Supplementary Information:**

The online version contains supplementary material available at 10.1186/s12859-022-04867-9.

## Background

In the past decades, many RNAi therapeutic programs focusing on cancer, metabolic diseases, respiratory disorders, retinal degeneration, dominantly inherited brain, skin diseases and infectious diseases had entered the clinical practice [1, 2], several RNAi based antiviral therapeutic projects had also reached at clinical trial stages [3, 4]. More recently, some researchers reported the identification of a group of endogenous siRNAs that played a part in enhancing environmental stress responses by repressing translation [5, 6]. However, the gene silencing effectiveness of RNAi relied on the siRNA efficacy in targeting a specific gene, so the efficacy prediction method constituted a huge challenge in selecting the potent siRNAs [7]. In general, researchers mainly used the machine-learning algorithms to design potent siRNAs [8–10], and focused on these features that contained empirical rules [11, 12], nucleotide frequency [10], binary pattern [13, 14], thermal stability [13], and many hybridized approaches [10].

However, for these commonly used features [10–14], there were no directly experimental evidences showing that they were able to influence siRNA activity [7], so their reliability needed to be validated when they were used to define the similarity of siRNAs. Here, *MG*-algorithm and *Icc*-cluster were used to verify their reliability, where *MG*-algorithm was able to generate such mini-groups that their samples were the nearest neighbors with each other [15], and *Icc*-cluster was able to put the distant samples to the different mini-clusters [16]. Results showed that most potent siRNAs of test data were unable to search their nearest neighbors from potent ones of training data.

Moreover, for theses commonly used algorithms for selection of potent siRNAs, they tried to constructing the overall difference between potent and ineffective siRNAs, such as ThermoComposition-21 [17], DSIR11 [18], i-score [19] that were both in the classification and regression modes, Biopredsi [20] that tried to combine the features together with the rules as input, ANN [20] that used two kinds of siRNA sequence features as feature set, Linear [21] that was linear regression model that was constructed by nucleotide preference scores, and SVM [7, 14, 17] that based on deep learning algorithm. However, potent and ineffective siRNAs belonged to a chaotic system when their similarity were defined by these commonly used features. Thus, for any of these algorithms, it might misidentify many ineffective siRNAs when it tried to searching the majority of potent ones.

Here, we firstly constructed *C*-features of siRNAs by *MG*-algorithm, *Icc*-algorithm and the hybridized features of these commonly used features, where these hybridized features were the different combinations of the frequencies of multi-nucleotides and the binary codings of their sequences. Then, for these ineffective siRNAs of test data, they were continually removed from test data and put to training data by *I*-iteration, where *I*-iteration continually updated training data by these successively removed siRNAs. In this study, for any removed siRNA of test data, its overall similarity with ineffective siRNAs of training data exceeded all potent siRNAs of training data. Moreover, we used Hencken dataset [7] to validate the reliability of our algorithm. For siRNAs of test data, results showed that our algorithm was able to remove the ineffective siRNAs from test data, gave the clear boundary between their potent and ineffective ones, and also accurately predicted their efficacy. We hoped our algorithm was able to help the researchers to select the best effective siRNAs for use as potential therapeutics against important human viruses.

## Results

### Constructing training and test data

Hencken dataset contained over 1358 siRNA sequences targeting different human viruses and HIV siRNA database [5], where the experimental indicators of siRNAs were provided, the lengths of siRNAs were 19 bp, and 70% targeted gene knockdown was considered as the threshold to define potent and ineffective siRNAs.

In this paper, siRNAs of the data set were reordered by their observed inhibitions, and then these 20% siRNAs whose new serial numbers were multiple of 5 were selected to construct test data. That is, we selected 103 potent and 242 ineffective siRNAs to construct test data, and other 1380 siRNAs to construct training data.

Moreover, 20 test sets were randomly generated from Hencken dataset also, where we used test-*i* to denote the *i*-th test set, and test-*i* contain 103 potent and 242 ineffective siRNAs also. Here, the average identification results of these 20 test-*i* sets were used to compare the different algorithms, and the results of test data to show the details of our algorithm.

### Comparison of different $C_{k}$-features

**Fig. 1:**
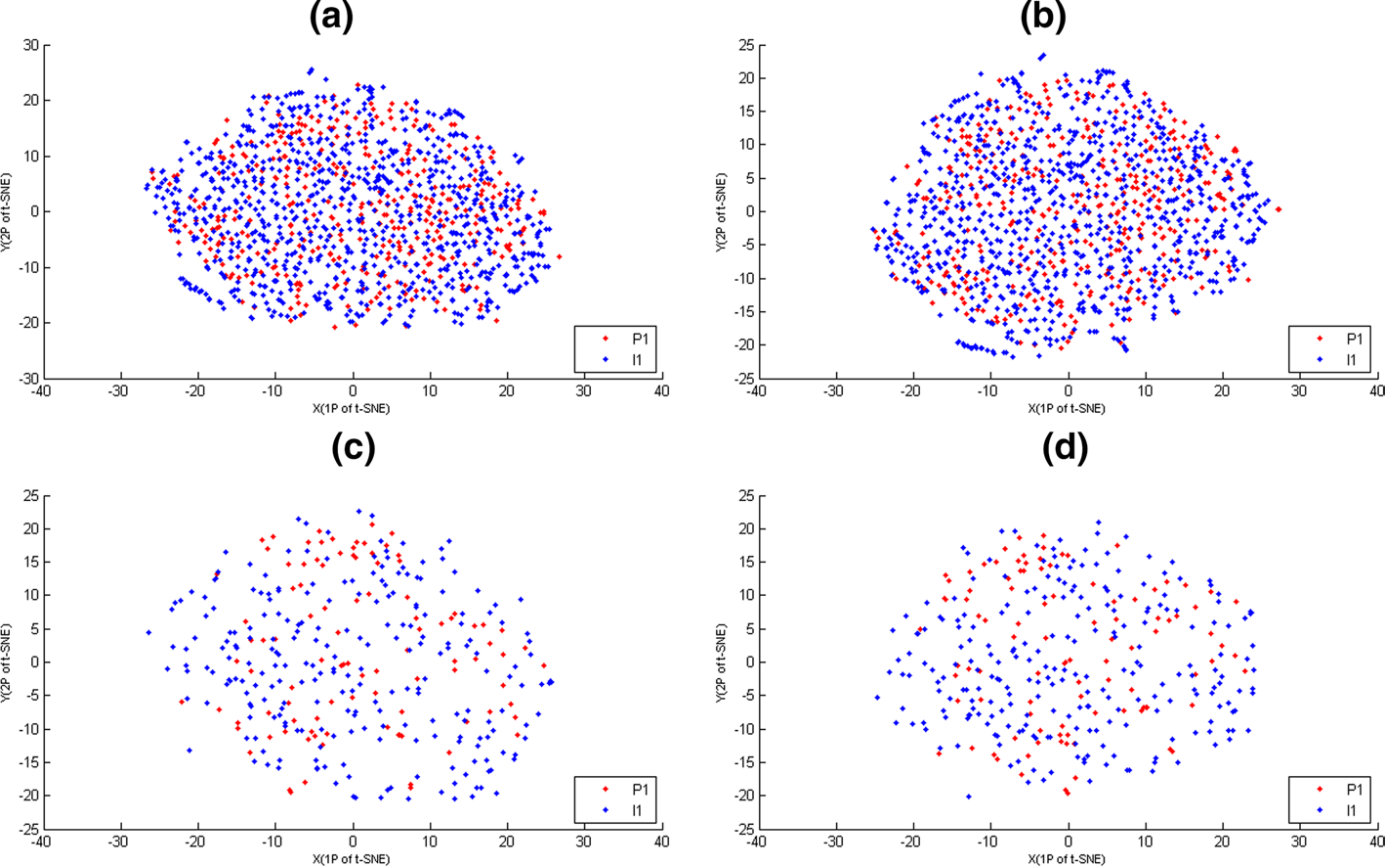
The t-SNE maps of siRNAs of training and test data, where potent and ineffective siRNAs were coloured according to their memberships. The X-axis represented the first projections (1P) of t-SNE. The Y-axis represented the second projections (2P) of t-SNE. **a** The t-SNE maps of $C_{15}$-features of training data. **b** The t-SNE maps of $C_{31}$-features of training data. **c** The t-SNE maps of $C_{15}$-features of test data. **d** The t-SNE maps of $C_{31}$-features of test data

Here, for siRNAs of training and test data, their $C_{15}$-features and $C_{31}$-features were displayed on t-SNE maps (Fig. 1) respectively, where t-SNE(t-statistic stochastic neighbor embedding) was a non-linear dimension reduction method which had been used to preserve local structure in the data [22], $C_{15}$-feature was the combination of 4 $F_{m}$-features, and $C_{31}$-feature was the combination of 4 $F_{m}$-features and *B*-feature. From Fig. 1, the potent and ineffective siRNAs were significantly intermixed with any $C_{k}$-features. That is, potent and ineffective siRNAs belonged to a chaotic system when their similarity were defined by $C_{k}$-features.

In fact, none of the commonly used features was able to give the clear boundary between potent and ineffective siRNAs, such as empirical rules [11, 12], nucleotide frequency [10], binary pattern [13, 14], thermal stability [13], and many hybridized approaches [10]. However, when we enlarged Fig. 1, we was able to find that some ineffective siRNAs were the nearest neighbors with each other. Thus, MG-algorithm(or *Icc*-cluster) with $C_{k}$-features was able to generate such mini-groups(or mini-clusters) that did not contain potent siRNAs of training data.

### Comparison of $C_{k}^{\alpha _{s},t}$-features and $D_{k}^{\alpha _{s},t}$-features

In this study, $C_{k}^{\alpha _{s},t}$-features and $D_{k}^{\alpha _{s},t}$-features were not used to removed ineffective siRNAs from test data. In fact, two elements of any of $C_{k}^{\alpha _{s},t}$-features had at most one 1, and the sum of three elements of any of $D_{k}^{\alpha _{s},t}$-features was 1.

However, for the fixing *k* and $\alpha _{s}$, $D_{k}^{\alpha _{s},t}(t=1,2,3,4)$-features of *R* had significant difference. The reason was that $C_{k}$-features did not follow the normal distribution, mini-groups of $MG_{1}$-algorithm, $MG_{2}$-algorithm, mini-clusters of $Icc_{1}$-cluster and $Icc_{2}$-cluster had significant difference.

Furthermore, since the goal of *I*-iteration was that continually removed ineffective siRNAs by $\alpha _{s}$-parameters, $C_{k}^{\alpha _{s},t}$-features were constructed by the first and third elements of $D_{k}^{\alpha _{s},t}$-features only.

### Comparison of different $C^{\alpha _{s},t}$-features

**Fig. 2:**
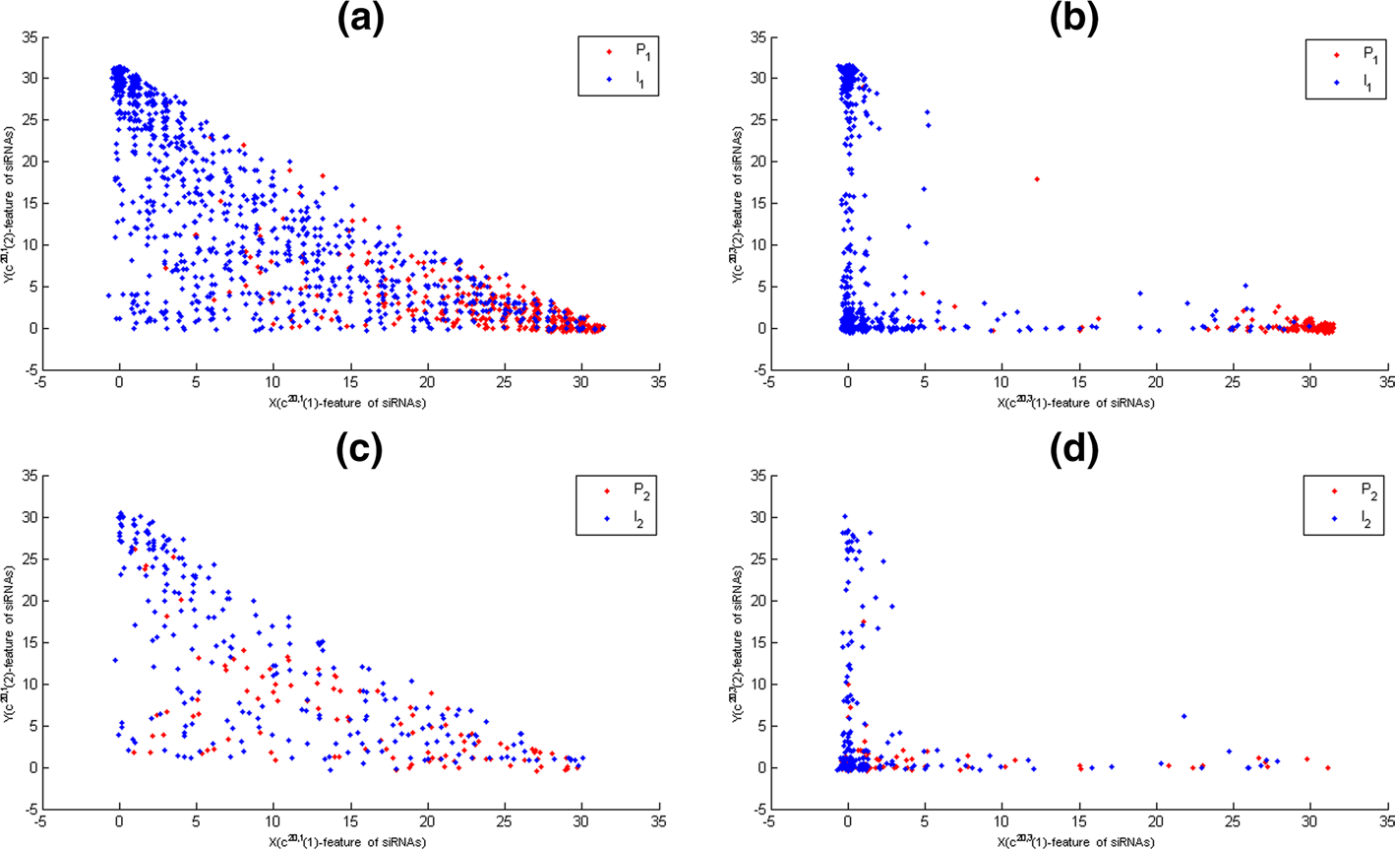
The $C^{\alpha _{s},t}$-feature maps of training and test data, where potent and ineffective siRNAs were coloured according to their memberships. The X-axis represented $c^{20,t}(1)$-features of siRNAs. The Y-axis represented $c^{20,t}(2)$-features of siRNAs. **a** The map of $C^{20,1}$-features of siRNAs of training data. **b** The map of $C^{20,3}$-features siRNAs of training data. **c** The map of $C^{20,1}$-features of siRNAs of test data. **d** The map of $C^{20,3}$-features siRNAs of test data

Here, for siRNAs of training and test data, their $C^{20,1}$-features and $C^{20,3}$-features were directly mapped on Fig. 2 by their two elements, respectively. Figure 2 showed that $C^{20,1}$-features and $C^{20,3}$-features were not able to give the clear boundary between potent and ineffective siRNAs, but they had a tendency to separate potent and ineffective siRNAs. Importantly, for the second elements of $C^{20,1}$-features and $C^{20,3}$-features of siRNAs, Fig. 2 showed that the largest ones came some ineffective siRNAs of training and test data at the same time. Thus, $C^{\alpha _{s},t}$-features could be used to remove some ineffective siRNAs from test data. Moreover, Fig. 2 showed that $C^{20,1}$-features and $C^{20,3}$-features had significant difference also.

### The reliability of *I*-iteration

**Fig. 3:**
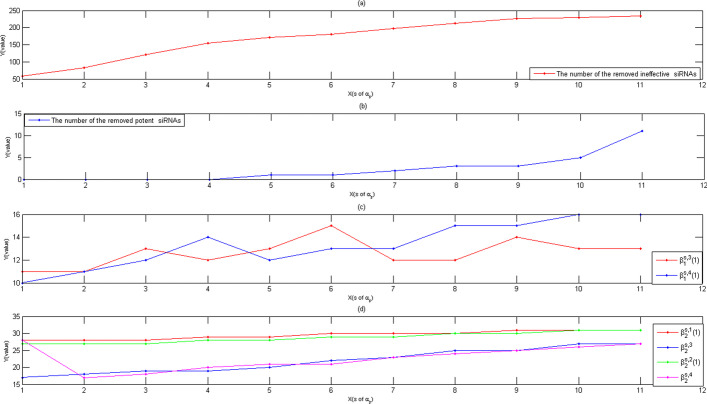
**a** The cumulative number of the removed ineffective siRNAs, where the X-axis represented $\alpha _{s}$, the Y-axis represented the number of the removed siRNAs. **b** The cumulative number of the removed potent siRNAs, where the X-axis represented $\alpha _{s}$, the Y-axis represented the number of the removed siRNAs. **c** The map of $\beta ^{s,3}_{1}(1)$-parameters and $\beta ^{s,4}_{1}(1)$-parameters. **d** The map of $\beta ^{s,t}_{2}(1)$-parameters

Here, for *I*-iteration with $\alpha _{s}$-parameters, the cumulative numbers of their removed ineffective and potent siRNAs were mapped on Fig. 3a and b, respectively. From Fig. 3a and b, when $\alpha _{s}$-parameter was less than 70%, *I*-iteration removed few potent siRNAs, and almost ineffective ones from test data. However, when $\alpha _{s}$-parameter was equal to 70%, *I*-iteration removed 10 potent siRNAs from test data. In fact, for some siRNAs that their efficacy was from 65 to 75%, their $C^{\alpha _{s},t}$-features had no significant difference. To prevent that *I*-iteration falsely removed potent siRNAs from test data, 65% was selected as the largest $\alpha _{s}$-parameter.

Moreover, in all removals of *I*-iteration, we found all $\beta ^{s,1}_{1}(1)$-parameters and $\beta ^{s,2}_{1}(1)$-parameters were equal to zero, where we only showed $\beta ^{s,t}(1)$-parameters that were the first constructed by $\alpha _{s}$-parameters. That is, for siRNAs of test data, these ones were removed from test data that all their $c^{\alpha ,1}(1)$-features(or $c^{\alpha ,2}(1)$-features) were zero.

Furthermore, all $\beta ^{s,3}_{1}(1)$-parameters and $\beta ^{s,4}_{1}(1)$-parameters were mapped on Fig. 3c, respectively, Fig. 3c showed that all $\beta ^{s,3}_{1}(1)$-parameters and $\beta ^{s,4}_{1}(1)$-parameters were greater than 10. That is, for these siRNAs of test data that their $c^{\alpha ,3}(1)$-features(or $c^{\alpha ,4}(1)$-features) were zero, their $c^{\alpha ,3}(2)$-features(or $c^{\alpha ,4}(2)$-features) were greater than 10 might be removed from test data.

At last, Fig. 3d showed that $\beta ^{s,t}_{2}(1)(t=1,2)$-parameters were greater than 27, while $\beta ^{s,t}_{2}(1)(t=3,4)$-parameters were relatively small.

That is, for any removed siRNAs of test data, its overall similarity with ineffective siRNAs of training data exceeded all potent siRNAs of training data.

### The boundary between potent and ineffective siRNAs

**Fig. 4:**
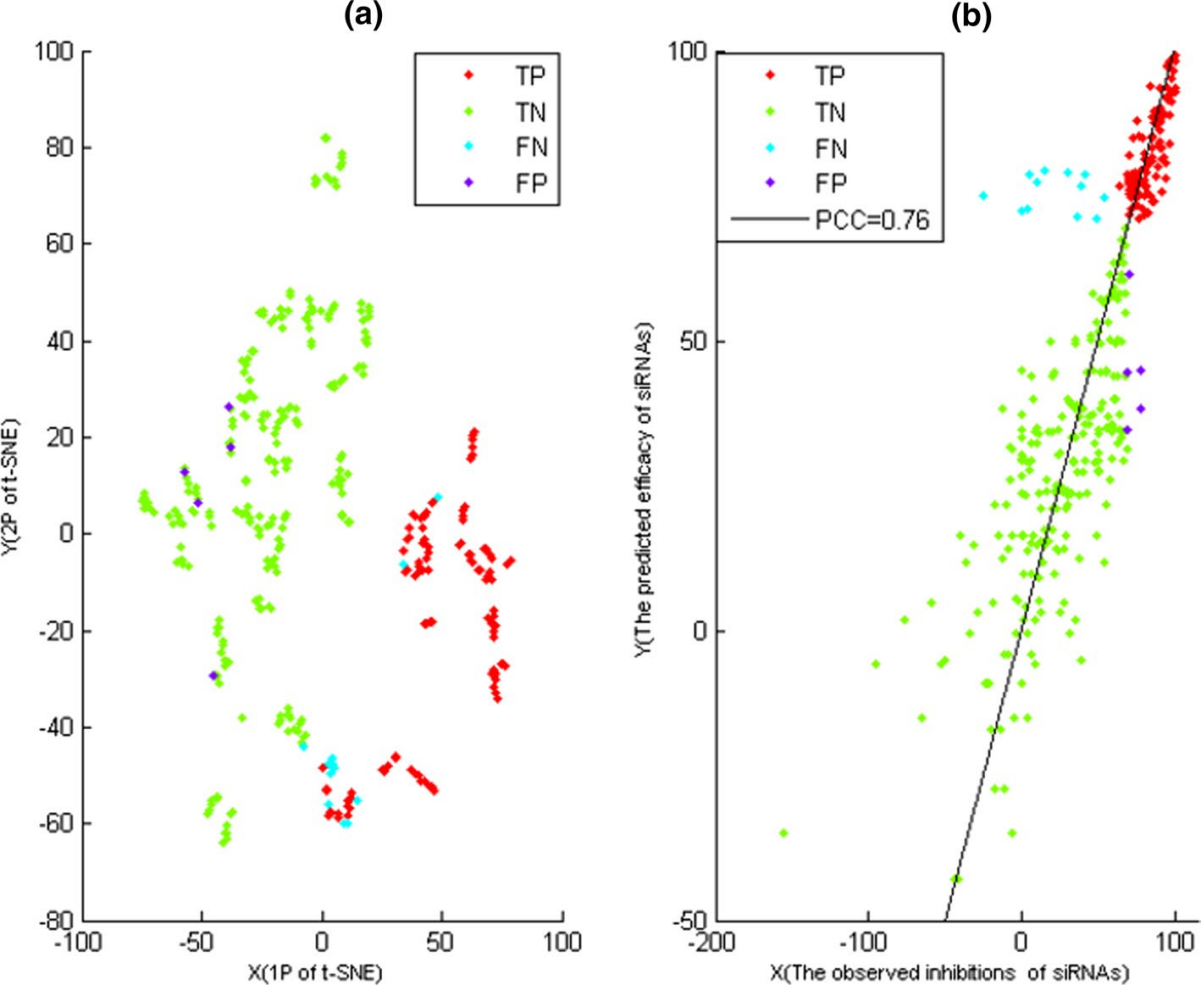
**a** The t-SNE map of $C^{{\alpha _{10}}}$-features of siRNAs in test data, where the X-axis represented the first projections (1P) of t-SNE, the Y-axis represented the second projections (2P) of t-SNE, *TN*, *FN*, *TP* and *FP* of siRNAs were coloured according to their memberships. **b** The predicted efficacy and observed inhibition of siRNAs, where *TN*, *FN*, *TP* and *FP* of siRNAs were coloured according to their memberships, the X-axis represented the observed inhibition of siRNAs, and the Y-axis represented the predicted efficacy of siRNAs

Here, for potent and ineffective siRNAs of test dat, their boundary were constructed by $C^{{\alpha _{10}}}$-features( Eq. 7), where their $C^{{\alpha _{10}}}$-features were displayed on t-SNE map(Fig. 4a), and $C^{{\alpha _{10}}}$-features were generated from the updated training data of *I*-iteration. Fig. 4a showed that $C^{{\alpha _{10}}}$-features gave the relatively clear boundary between potent and ineffective siRNAs of test data.

### The distinguishing results of *P*-cluster and *I*-cluster

**Table 1:** The distinguishing results of siRNAs

Data	Tool	Se (%)	Sp (%)
Test data	*I*-iteration	87.5	97.9
20 test-*i* sets	*I*-iteration	81.5*	96.6*
20 test-*i* sets	Score-Level	63.1*	92.2*
20 test-*i* sets	ThermoComposition-21	51.9*	89.9*
20 test-*i* sets	DSIR	49.2*	88.9*
20 test-*i* sets	i-score	48.2*	88.3*
20 test-*i* sets	Biopredsi	46.9*	87.4*

Column headers are defined as the same paper

*Is the average value of Se(or Sp) of these 20 test-*i* sets

**Table 2:** The results of *TN*,*FN*, *TP* and *FP* of different test sets

Data	Algorithm	*TN*	*FN*	*TP*	*FP*
Test data	*I*-iteration	228	14	98	5
20 test-*i* sets	*I*-iteration	210.3*	21.7*	95.5*	7.5*
20 test-*i* sets	Score-Level	180.7*	51.3*	87.7*	15.3*
20 test-*i* sets	ThermoComposition-21	152.4*	79.6*	85.8*	17.2*
20 test-*i* sets	DSIR	145.8*	86.2*	83.5*	19.5*
20 test-*i* sets	i-score	141.3*	90.7*	84.3*	18.7*
20 test-*i* sets	Biopredsi	137.9*	94.1*	83.2*	19.8*
test-a data	*I*-iteration	231	11	99	4
test-b data	*I*-iteration	235	7	96	7
test-c data	*I*-iteration	218	24	100	3
test-d data	*I*-iteration	224	18	97	6

Column headers are defined as the same paper

*Are the average value of *TN*,*FN*, *TP* and *FP* of these 20 test-*i* sets, respectively

For siRNAs of test data, their distinguishing results of *P*-cluster and *I*-cluster were summarized in Tables 1 and 2. From Table 2, *TN*, *FN*, *TP* and *FP* of test data were 228, 14, 98 and 5 respectively. That is, only 5 potent and 14 ineffective siRNAs of test data were misidentified, respectively. Moreover, the distinguishing result of test data was displayed on t-SNE map by $C^{{\alpha _{10}}}$-features of siRNAs (Fig. 4a). Furthermore, Fig. 4a was able to help us to search these misidentified siRNAs.

Moreover, for *TN*, *FN*, *TP* and *FP* of 20 test-*i* sets, their average value were 210.3, 21.7, 95.5 and 7.5 respectively, and the details were summarized in the second row of Table 2. That is, the average distinguishing results of 20 test-*i* sets were slightly less compared to ones of test data. The reason was that some ineffective siRNAs were easier to search their neighbors from these ones with similar efficiency. These results demonstrated that *I*-iteration was able to correctly remove ineffective siRNAs from test data.

### Predicting efficacy of siRNAs

Here, for siRNAs of test data, their efficacy were predicted by Eq. (10), where the predicting results were summarized in Fig. 4b and Table 2. Table 2 showed that PCC of the predicting efficacy was equal to 0.76 that was calculated by Eq. (12). Moreover, for 20 test-*i* sets, the average value of their PCCs was equal to 0.73 (Table 2). That is, the average PCCs of 20 test-*i* sets were slightly less than one of test data.

And more importantly, Fig. 4b showed that the efficacy of siRNAs in *P*-cluster (or *I*-cluster) was greater (or less) than 70%. This was because *I*-iteration gave the relatively clear boundary between *P*-cluster and *I*-cluster. That is, for almost potent(or ineffective) siRNAs of test data, their predicting efficacy of Eq. (10) were potent (or ineffective) also.

### Comparison to existing design algorithms

For the distinguishing results of Score-Level [7], ThermoComposition-21 [17], DSIR11 [18], i-score [19] and Biopredsi [20], they were summarized in Tables 1 and 2, where these results were the average value of these 20 test-*i* sets, Score-Level used F-score to investigate the contribution of each feature and remove the weak relevant features to SVM [7], ThermoComposition-21 combined position features and thermodynamic features to an artificial neural network model [17], DSIR11 used basic sequence information and a simple linear model LASSO [18], i-score utilized linear regression models to perform art-of-the-state accuracy rates [19], and Biopredsi applied artificial neural networks to predict siRNA efficacy [20]. Table 1 showed that the highest sensitivity of those servers came from Score-Level [7] that was 63.1% only. Moreover, Table 2 showed that the poor sensitivity of those servers was generated from the large *FN*. For instance, for DSIR11, i-score and Biopredsi, their *FN* were greater than their *TP*. That is, the numbers of their misidentified ineffective siRNAs were greater than their correctly identified potent ones. In fact, these algorithms tried to constructing the overall difference between potent and ineffective siRNAs, but siRNAs belonged to a chaotic system when their similarity were defined by these commonly used features. Thus, for any of these algorithms, it might misidentify many ineffective siRNAs when it tried to searching the majority of potent ones. Furthermore, Tables 1 and 2 showed that the distinguishing results of hybridized features (Score-Level and ThermoComposition-21) were superior to ones of relatively simple features(DSIR11), and the nonlinear results(Score-Level [7] and ThermoComposition-21) were superior to linear ones also(DSIR11 and i-score). In total, these results verified that these algorithms were unable to construct the clear boundary between potent and ineffective siRNAs.

Compared to above algorithms, the sensitivity of *I*-iteration(81.5%) was far more than any one of them. The reason was that *FN* of *P*-cluster and *I*-cluster was far less than ones of other algorithms. In fact, *I*-iteration was used to remove ineffective siRNA from test data, and only these ones that their overall similarity with ineffective siRNAs of training data exceeded all potent siRNAs of training data were removed from test data. And more importantly, *I*-iteration did not construct the overall difference between potent and ineffective siRNAs, it only continually updated training data by these successively removed siRNAs.

**Table 3:** The efficacy prediction results of siRNAs

Data	Tool	Algorithm	PCC
Test data	Eq. (12)	Eq. (12)	0.76
20 test-*i* sets	Eq. (12)	Eq. (12)	0.73*
20 test-*i* sets	Biopredsi [20]	ANN	0.62*
20 test-*i* sets	[21]	Linear	0.58*
20 test-*i* sets	ThermoComposition-21 [17]	SVM	0.71*
20 test-*i* sets	[14]	SVM	0.55*
20 test-*i* sets	Score-Level [7]	SVM	0.73*

Column headers are defined as the same paper

*Is the average value of PCCs value of these 20 test-*i* sets

Here, the efficacy predicting results of ANN [20], Linear [21] and SVM [7, 14, 17] were summarized in Table 3, where ANN used the artificial neural network to train on a complementary 21-nucleotide guide sequence [20], Linear used support vector machine regression by combining and filtering features [21], SVM [14] used various characteristic methods, and SVM [17] used thermodynamic and composition features. From Table 3, the highest PCC of these servers came from Score-Level and ThermoComposition-21(SVM [14]) also. That is, the better efficacy prediction was generated from the better classification.

Compared to above algorithms, the efficacy prediction of Eq. (10) was nearly equal to Score-Level, but the classification of *I*-iteration was far more than Score-Level. The reason was that $C^{\alpha _{10}}$-features only constructed the overall difference between potent and ineffective siRNAs.

### The sensitivity analysis of our algorithm

In the process of constructing features, $\alpha _{s}$-parameters were beginning with 20%, and ending with 65%. Naturally, this begged a follow-up question, that is, whether similar distinguishing results of our algorithm could be constructed by other initial and final values. In fact, for $\alpha _{s}$-parameters that were beginning with 10% and ending with 65%, their distinguishing results had no difference compared to our used values. That is, *P*-cluster and *I*-cluster were not sensitive to the initial values of $\alpha _{s}$-parameters.

### The cross-validation of our algorithm

Here, we also used these siRNAs whose new serial numbers were multiple of 1 (or 2, or 3, or 4) to construct test-a(or test-b, or test-c, or test-d) data, and other siRNAs to construct training data. Then, their *P*-cluster and *I*-cluster were constructed by Eq. (10), and the distinguishing results were summarized in Table 2. Table 2 showed that the distinguishing result of 5 groups of *P*-clusters and *I*-clusters had no difference compared to our used test data. These results demonstrated that ineffective siRNAs were easier to search their neighbors from these ones with similar efficiency, and our algorithm was able to ensure non-randomness of the performance in experiments also.

## Discussion

In fact, *MG*-algorithm (or *Icc*-cluster) with $C_{k}$-features is able to produce some these ineffective mini-groups(or mini-clusters) that do not contain potent ones of training data, where these ineffective mini-groups(or mini-clusters) contain about 20% siRNAs of test data. That is, for some ineffective siRNAs of test data, they are relatively easy to search their nearest neighbors from ineffective ones of training data. That is, some ineffective siRNAs exist local similarity with $C_{k}$-features. And more importantly, for different $C_{k}$-features, their ineffective mini-groups(or mini-clusters) have significant difference. Thus, if we construct enough these ineffective mini-groups(or mini-clusters) that have significant difference, we are able to remove ineffective siRNAs from test data.

Moreover, for most $C^{65,t}$-features that are constructed by raw training data, they can remove more than 70% ineffective siRNAs from test data, but a penalty to be paid for about 20% potent siRNAs of test data are falsely removed also. To remain potent siRNAs in test data, *I*-iteration uses $\beta ^{s,t}$-parameters that only removed ineffective siRNAs from test data. In fact, the conditions of $\beta ^{s,t}$-parameters for these removal are very harsh. That is, for any removed siRNAs of test data, its overall similarity with ineffective siRNAs of training data exceeds all potent siRNAs of training data. In fact, we can only remove about 35% of ineffective siRNAs of test data at a time, so we use *I*-iteration to construct 10 removals.

Since 70% targeted gene knockdown is considered as the threshold to define potent and ineffective siRNAs, 65% is selected as the largest $\alpha _{s}$-parameter. This prevents that potent siRNAs are falsely removed from test data by *I*-iteration. Furthermore, since the number of siRNAs in training data is selected as the clustering number of *Icc*-cluster, $C^{\alpha _{s},3}$-features and $C^{\alpha _{s},4}$-features of potent siRNAs in training data have significant advantage compared to ones in test data. This ensures that *I*-iteration does not remove potent siRNAs from test data also.

## Conclusion

In fact, the key to success of our algorithm is *MG*-algorithm, which does not focus on searching the overall difference between potent and ineffective siRNAs, but constructs the local similarity of ineffective ones. That is, if some ineffective siRNAs are highly correlated with some specific features, *MG*-algorithm can extract their similarity by mini-groups. In total, we hope our algorithm can be useful in predicting highly potent siRNAs to aid therapeutic development.

## Methods

Here, for siRNAs of training data, we use *X*(*i*) and *Y*(*j*) to denote the *i*-th and *j*-th potent and ineffective ones, respectively. Moreover, we use *Z*(*l*) to denote the *l*-th siRNA of test data, where the efficacy of *Z*(*l*) is seen as unknown.

### $C_{k}$-features of siRNAs

For *R* that is a random siRNA, its $F_{1}$-feature, $F_{2}$-feature, $F_{3}$-feature and $F_{4}$-feature are constructed by the frequencies of mononucleotide, dinucleotide, trinucleotide and tetranucleotide of the sequence of $R_{m}$ respectively, where

1 $$\begin{aligned} \left\{ \begin{array}{lll} R&{}=&{}X(i), \text{ or } Y(j), \text{ or } Z(l)\\ R_{m}&{}=&{}Rr_{1}\cdots r_{m-1},~r_{s} \text{ is } \text{ the } \text{ nucleotide } \text{ of } \text{ R } \text{ in } \text{ the } \text{ s-th } \text{ position } \\ F_{m}&{}=&{}\{f_{m}(1), f_{m}(2),\cdots ,f_{m}(4^{m})\},~ \sum _{l=1}^{4^{m}}f_{m}(l)=19 \end{array} \right. \end{aligned}$$

Moreover, *B*-feature of *R* is constructed by its binary codings of nucleotide, where

2 $$\begin{aligned} \left\{ \begin{array}{lll} B&{}=&{}\{b(1), b(2),\cdots ,b(76)\}\\ b(l)&{}=&{}0,1, l=1,2,\cdots ,76\\ \sum _{l=4t+1}^{4t+4}b(l)&{}=&{}1,t=0,1,\cdots , 18,~ \sum _{l=1}^{76}b_{m}(l)=19 \end{array} \right. \end{aligned}$$

Furthermore, 31 $C_{k}$-features of *R* are constructed by the different combinations of 4 $F_{m}$-features and 1 *B*-feature. That is, any $C_{k}$-feature contains one or more $F_{m}$-features and *B*-feature.

### *MG*-algorithm and *Icc*-cluster

*MG*-algorithm directly puts the nearest neighbor siRNAs in the same mini-groups [15]. That is, when a siRNA belongs to a mini-group, its nearest neighbor is also in the mini-group, where $MG_{1}$-algorithm and $MG_{2}$-algorithm use Euclidean distance and PCC (Pearson Correlation Coefficient) to define the similarity of siRNAs, respectively. But for *Icc*-cluster algorithm, its clustering centers are generated from these most distant siRNAs with each other, and other siRNAs are put to mini-clusters by searching their nearest centers [16], where $Icc_{1}$-cluster and $Icc_{2}$-cluster use Euclidean distance and PCC to define the similarity of siRNAs, respectively. Moreover, the freely available MATLAB implementes to perform *MG*-algorithm and *Icc*-cluster are summarized in Additional file 1.

In fact, for many potent siRNAs of test data, their nearest neighbors come from ineffective ones of training data. To separate these nearest neighbors that come from different efficient categories, the number of siRNAs of training data is selected as the clustering number of *Icc*-cluster. Results show that some of these nearest neighbor siRNAs are put to different mini-clusters by *Icc*-cluster with the clustering number.

### $\alpha _{s}$-parameters

Here, siRNAs of training data are specified as 3 *E*-groups by a $\alpha _{s}$-parameter, where

3 $$\begin{aligned} \left\{ \begin{array}{l} \alpha _{s}=(20+5(s-1))\%, s=1,2,\cdots ,11\\ X(i)\in E_{1}\\ Y(j)\in E_{2}, \alpha _{s}\le Y_{e}(j)<70\%\\ Y(j)\in E_{3}, Y_{e}(j)< \alpha _{s} \end{array} \right. \end{aligned}$$

$Y_{e}(j)$ is the experimental efficacy of *Y*(*j*), and $\alpha _{s}$-parameter is a artificial efficacy boundary between ineffective siRNAs.

### $D_{k}^{\alpha _{s},t}$-features of siRNAs

For siRNAs of training and test data, $MG_{1}$-algorithm with their $C_{k}$-features divides them into mini-groups, where $G_{k}^{u}$-group is used to denote the *u*-th mini-group. For *R*, if it is put to $G_{k}^{p}$-group, its $D_{k}^{\alpha _{s},1}$-feature is constructed, where

4 $$\begin{aligned} \left\{ \begin{array}{lll} D_{k}^{\alpha _{s},1}&{}=&{}\{d_{k}^{\alpha _{s},1}(1),d_{k}^{\alpha _{s},1}(2),d_{k}^{\alpha _{s},1}(3)\}\\ d_{k}^{\alpha _{s},1}(1)&{}=&{}\frac{\text{ N } \{G_{k}^{p}\bigcap E_{1}\}}{\text{ N } \{G_{k}^{p}\}}\\ d_{k}^{\alpha _{s},1}(2)&{}=&{}\frac{\text{ N } \{G_{k}^{p}\bigcap E_{2}\}}{\text{ N } \{G_{k}^{p}\}}\\ d_{k}^{\alpha _{s},1}(3)&{}=&{}\frac{\text{ N } \{G_{k}^{p}\bigcap E_{3}\}}{\text{ N } \{G_{k}^{p}\}} \end{array} \right. \end{aligned}$$

$N\{G_{k}^{p}\bigcap E_{l}\}$ is the siRNA number of the intersection of $G_{k}^{p}$ and $E_{l}(l=1,2,3)$, and $N\{G_{k}^{p}\}$ is the siRNA number of $G_{k}^{p}$-group.

### $C_{k}^{\alpha _{s},t}$-features of siRNAs

Here, $C_{k}^{\alpha _{s},1}$-feature of *R* is constructed by its $D_{k}^{\alpha _{s},1}$, where

5 $$\begin{aligned} \left\{ \begin{array}{lll} C_{k}^{\alpha _{s},1}&{}=&{}\{c_{k}^{\alpha _{s},1}(1),c_{k}^{\alpha _{s},1}(2)\}\\ c_{k}^{\alpha _{s},1}(1)&{}=&{}\left\{ \begin{array}{lll}0,d_{k}^{\alpha _{s},1}(1)\le d_{k}^{\alpha _{s},1}(3)\\ 1, d_{k}^{\alpha _{s},1}(1)> d_{k}^{\alpha _{s},1}(3) \end{array}\right. \\ c_{k}^{\alpha _{s},1}(2)&{}=&{}\left\{ \begin{array}{lll}0,d_{k}^{\alpha _{s},1}(1)\ge d_{k}^{\alpha _{s},1}(3)\\ 1, d_{k}^{\alpha _{s},1}(1)< d_{k}^{\alpha _{s},1}(3) \end{array}\right. \\ \end{array} \right. \end{aligned}$$

Moreover, $C_{k}^{\alpha _{s},2}$-features, $C_{k}^{\alpha _{s},3}$-features and $C_{k}^{\alpha _{s},4}$-features of siRNAs are constructed by $MG_{2}$-algorithm, $Icc_{1}$-cluster and $Icc_{2}$-cluster respectively, where *k* is from 1 to 31, *s* is from 1 to 11, and *t* is from 1 to 4.

### $C^{\alpha _{s},t}$-features of siRNAs

For *R*, its $C^{\alpha _{s},t}$-feature is constructed by its 31 $C_{k}^{\alpha _{s},t}$, where

6 $$\begin{aligned} \left\{ \begin{array}{lll} C^{\alpha _{s},t}&{}=&{}\{c^{\alpha _{s},1}(t),c^{\alpha _{s},t}(2)\},t=1,2,3,4\\ c^{\alpha _{s},t}(i)&{}=&{}\sum _{k=1}^{31}c_{k}^{\alpha _{s},t}(i), i=1,2 \end{array} \right. \end{aligned}$$

### $C^{\alpha _{s}}$-features of siRNAs

For *R*, its $C^{\alpha _{s}}$-feature is the combination of four types $C^{{\alpha _{s}},t}$-features, where

7 $$\begin{aligned} \begin{array}{l} C^{\alpha _{s}}=\{C^{\alpha _{s},1},C^{\alpha _{s},2},C^{\alpha _{s},3},C^{\alpha _{s},4}\} \end{array}.\end{aligned}$$

### *I*-iteration

Here, for ineffective siRNAs of test data, *I*-iteration uses $\beta ^{s,t}$-parameters to remove them from test data, where $\beta ^{s,t}$-parameters are constructed by $C^{\alpha _{s},t}$-features(Eq. 6) of *X*(*i*), and

8 $$\begin{aligned} \left\{ \begin{array}{l} \beta ^{s,t}=\{\beta ^{s,t}_{1}, \beta ^{s,t}_{2}\}\\ \beta ^{s,t}_{1}=\text{ min }\{c^{\alpha _{s},t}(1) \text{ of } X(i)\}, t=1,2\\ \beta ^{s,t}_{1}=\text{ max }\{c^{\alpha _{s},t}(2) \text{ of } \text{ these } X(i) \text{ that } \text{ their } c^{\alpha _{s},t}(1) \text{ is } \text{0 } \},t=3,4\\ \beta ^{s,t}_{2}=\text{ max }\{c^{\alpha _{s},t}(2) \text{ of } X(i)\} \end{array} \right. \end{aligned}$$

Then, for any *Z*(*l*) of test data, it is removed from test data if its $C^{\alpha _{s},t}$-feature satisfies any of the conditions of Eq. (9), where Eq. (9) is defined as

9 $$\begin{aligned} \left\{ \begin{array}{l} c^{\alpha _{s},t}(1)\le \beta ^{s,t}_{1}, t=1,2\\ c^{\alpha _{s},t}(2)\ge \beta ^{s,t}_{2}, t=1,2,3,4\\ c^{\alpha _{s},t}(1)=0, c^{\alpha _{1},t}(2)\ge \text{ min }\{\beta ^{s,t}_{1}, 16+s\}, t=3,4 \end{array} \right. \end{aligned}$$

In details, the iteration process is constructed by the following:

*Step* 1 Based on $\alpha _{1}$-parameter and $\beta ^{1,t}$-parameters that are constructed by Eq. (8), these siRNAs of test data are removed from test data if their $C^{\alpha _{1},t}$-features satisfy any of the conditions of Eq. (9), where $\alpha _{s}$ of Eq. (9) is $\alpha _{1}$. Moreover, the copies of these removed siRNAs are put to *I*-cluster and $E_{3}$-group(Eq. 3) simultaneously. That is, the training data is updated.

*Step* 2 Based on the updated training data of Step 1, Repeat Step 1 until no *Z*(*l*) can be removed from test data by new $\beta ^{1,t}$-parameters, where we obtain new $\beta ^{1,t}$-parameter by the updated training data of Step 1.

*Step* 3 Repeat Step 1 and 2 until $\beta ^{11,t}$-parameter does not remove *Z*(*l*) from test data.

That is, the updated training data stops until $\alpha _{11}=70$. At last, the remaind siRNAs of test data are put to *P*-cluster. Here, for siRNAs of test data, they are distinguished as potent(or ineffective) ones when they belong to *P*-cluster (or *I*-cluster).

### Predicting efficacy of siRNAs

Here, for siRNAs of *P*-cluster and potent ones of training data (or siRNAs of *I*-cluster and ineffective ones of training data), they are divided into mini-groups by $MG_{1}$-algorithm with their $C^{\alpha _{10}}$-features (Eq. 7), where $C^{{\alpha _{10}}}$-features are generated from the updated training data of *I*-iteration, the efficacy of *Z*(*l*) is predicted by Eq. (10),

10 $$\begin{aligned} \widehat{Z_{e}(l)} = \left\{ \begin{array}{lll}\frac{1}{u}\sum _{i=1}^{u} {X_{e}(i)}, Z(l) \in \text{ P-cluster }\\ \frac{1}{v}\sum _{j=1}^{v}{Y_{e}(j)}, Z(l) \in \text{ I-cluster } \end{array}\right. ,\end{aligned}$$

$\widehat{Z_{e}(l)}$ is the predicted efficacy of *Z*(*l*), $X_{e}(i)$(or $Y_{e}(j)$) is the experimental indicator of *X*(*i*)(or *Y*(*j*)), *u*(or *v*) is the number of potent (or ineffective) siRNAs of the mini-group that contains *Z*(*l*).

### Sensitivity and specificity

Here, we use *Se*(sensitivity) and *Sp*(specificity) to evaluate the consistency between the experiment indicators and clustering results, where the experimental indicators are seen as the golden standard of genes, and *Se* and *Sp* are defined as

11 $$\begin{aligned} \left\{ \begin{array}{l} Se= \frac{TP}{TP+FN} \\ Sp= \frac{TN}{TN+FP}\\ \end{array}, \right. \end{aligned}$$

where *TN*, *FN*, *TP* and *FP* are the number of true negatives, false negatives, true positives and false positives, respectively.

### PCC model

In general, PCC model is used to measure the correlation between the predicted efficacy and observed inhibition, where

12 $$\begin{aligned} PCC=\frac{1}{b-1}\sum \limits _{l=1}^{b}\left( \frac{\widehat{Z_{e}(l)}-\overline{Z_{p}}}{\sigma _{Z_{p}}}\right) \left( \frac{Z_{e}(l)-\overline{Z_{e}}}{\sigma _{Z_{e}}}\right) , \end{aligned}$$

*b* is the number of siRNAs of test data, $\widehat{Z_{e}(l)}$ and ${Z_{e}(l)}$ are the predicted value and observed label of *Z*(*l*), $\overline{Z_{p}}$ and $\sigma _{Z_{p}}$ are the mean and standard deviation of all $\widehat{Z_{e}(l)}$, $\overline{Z_{e}}$ and $\sigma _{Z_{e}}$ are the mean and standard deviation of all $Z_{e}(l)$, respectively.

## Supplementary Information

**Additional file 1**: MATLAB algorithm. A freely available MATLAB implemented to perform MG-algorithm and Icc-cluster for a data set.

## Data Availability

Hencken dataset: http://crdd.osdd.net/servers/virsirnapred/dataset.php?dataset=1723, *MG*-algorithm: https://bmcbioinformatics.biomedcentral.com/articles/10.1186/s12859-018-2495-5, *Icc*-algorithm: https://febs.onlinelibrary.wiley.com/doi/10.1002/2211-5463.12327

## Abbreviations

$F_{m}(m=1,2,3,4)$-feature: They are defined by Eq. (1)
*B*-feature: It is defined by Eq.(2)
$C_{k}$-features: They are constructed by the different combinations of its 4 $F_{m}$-features and 1 *B*-feature
$D_{k}^{\alpha _{s},t}$-feature of *R*: It is defined by Eq. (4)
$C_{k}^{\alpha _{s},t}$-feature of *R*: It is defined by Eq. (5)
$C^{\alpha _{s},t}$-feature of *R*: It is defined by Eq. (6)
$C^{\alpha _{s}}$-feature of *R*: It is defined by Eq. (7)
$\alpha _{s}$-parameter: It is defined by Eq. (3)
$\beta$-parameter: It is defined by Eq. (8)
$\{ \beta ^{s,t}_{1}, \beta ^{s,t}_{2}\}$-parameter: It is defined by Eq. (8)
*P*-cluster: Containing these siRNAs of test data that are distinguished as potent ones
PCC: Pearson Correlation Coefficient
