# Supplementary material for: Constructing the boundary between potent and ineffective siRNAs by MG-algorithm with C-features
Source: BMC Bioinformatics. 2022 Aug 13;23:337. doi: 10.1186/s12859-022-04867-9 (PMC9375269; doi:10.1186/s12859-022-04867-9)
Supplement: Supplementary file 1 — Additional file 1: MATLAB algorithm. A freely available MATLAB implemented to perform MG-algorithm and Icc-cluster for a data set. [file 12859_2022_4867_MOESM1_ESM.doc]

A freely available MATLAB implemented to perform MG-algorithm

for a data set.

clc

Z1=importdata('siaA.xlsx');

Z2=importdata('siaA2.xlsx');

V1 = Z1.Sheet1;

V2 = Z2.Sheet1;

V=[V2;V1;V2]

[s1,s2] = size(V);

s=1.25;

for i=1:s1

for j=1:s2

idx1(i,j)=strfind(V(i,j),'A');

idx2(i,j)=strfind(V(i,j),'C');

idx3(i,j)=strfind(V(i,j),'G');

idx4(i,j)=strfind(V(i,j),'U');

end

end

xlswrite('g1A.xls',idx1);

xlswrite('g2A.xls',idx2);

xlswrite('g3A.xls',idx3);

xlswrite('g4A.xls',idx4);

R1=xlsread('g1A.xls');

R2=xlsread('g2A.xls');

R3=xlsread('g3A.xls');

R4=xlsread('g4A.xls');

R1(find(isnan(R1)==1)) = 0;

R2(find(isnan(R2)==1)) = 0;

R3(find(isnan(R3)==1)) = 0;

R4(find(isnan(R4)==1)) = 0;

ju00=[R1 R2 R3 R4];

t=ju00(2:s1-1,:)

[m,m1] = size(t);

n0=347;

n1=762;

n2=n1+1;

n3=m-1;

p1=1;

q1=19;

p2=q1+1;

q2=2*q1;

p3=2*q1+1;

q3=3*q1;

p4=3*q1+1;

q4=4*q1;

X = t;

Y1=X(:,p1:q1);

U1=Y1(n0:n1,:);

V1=Y1(n2:n3,:);

K1=mean(U1);

L1=mean(V1);

C1=L1./K1;

for i=1:m

for j=1:q1

if C1(1,j)>s

D1(i,j)=Y1(i,j);

else

D1(i,j)=0;

end

end

end

E1=sum(D1,2);

F1=sum(Y1,2);

G1=F1-E1;

Y2=X(:,p2:q2);

U2=Y2(n0:n1,:);

V2=Y2(n2:n3,:);

K2=mean(U2);

L2=mean(V2);

C2=L2./K2;

for i=1:m

for j=1:q1

if C2(1,j)>s

D2(i,j)=Y2(i,j);

else

D2(i,j)=0;

end

end

end

E2=sum(D2,2);

F2=sum(Y2,2);

G2=F2-E2;

Y3=X(:,p3:q3);

U3=Y3(n0:n1,:);

V3=Y3(n2:n3,:);

K3=mean(U3);

L3=mean(V3);

C3=L3./K3;

for i=1:m

for j=1:q1

if C3(1,j)>s

D3(i,j)=Y3(i,j);

else

D3(i,j)=0;

end

end

end

E3=sum(D3,2);

F3=sum(Y3,2);

G3=F3-E3;

Y4=X(:,p4:q4);

U4=Y4(n0:n1,:);

V4=Y4(n2:n3,:);

K4=mean(U4);

L4=mean(V4);

C4=L4./K4;

for i=1:m

for j=1:q1

if C4(1,j)>s

D4(i,j)=Y4(i,j);

else

D4(i,j)=0;

end

end

end

E4=sum(D4,2);

F4=sum(Y4,2);

G4=F4-E4;

H1=[E1 E2 E3 E4];

H2=[F1 F2 F3 F4];

H3=[G1 G2 G3 G4];

H4=[D1 D2 D3 D4];

H5=X-H4;

xlswrite('h11.xls',H1);

xlswrite('h14.xls',H4);

tic

clc

clear all

q3=1726;

q4=416;

q6=346;

Z1=xlsread('h11.xls');

Z2=xlsread('h14.xls');

Z3=xlsread('h21.xls');

Z4=xlsread('h24.xls');

Z5=xlsread('h31.xls');

Z6=xlsread('h34.xlsx');

Z7=xlsread('h41.xls');

Z8=xlsread('h44.xlsx');

Y1=[Z1 Z3];

Y2=[Z1 Z4];

Y3=[Z1 Z5];

Y4=[Z1 Z6];

Y5=[Z1 Z7];

Y6=[Z1 Z8];

Y7=[Z2 Z3];

Y8=[Z2 Z4];

Y9=[Z2 Z5];

Y10=[Z2 Z6];

Y11=[Z2 Z7];

Y12=[Z2 Z8];

Y13=[Z3 Z5];

Y14=[Z3 Z6];

Y15=[Z3 Z7];

Y16=[Z3 Z8];

Y17=[Z4 Z5];

Y18=[Z4 Z6];

Y19=[Z4 Z7];

Y20=[Z4 Z8];

Y21=[Z5 Z7];

Y22=[Z5 Z8];

Y23=[Z6 Z7];

Y24=[Z6 Z8];

P1=Z1;

P2=Z2;

P3=[Z1 Z2];

P4=Z3;

P5=Z4;

P6=[Z3 Z4];

P7=Z5;

P8=Z6;

P9=[Z5 Z6];

P10=Z7;

P11=Z8;

P12=[Z7 Z8];

Q1=[Z1 Z2 Z3];

Q2=[Z1 Z2 Z4];

Q3=[Z1 Z2 Z5];

Q4=[Z1 Z2 Z6];

Q5=[Z1 Z2 Z7];

Q6=[Z1 Z2 Z8];

Q7=[Z1 Z3 Z4];

Q8=[Z1 Z3 Z5];

Q9=[Z1 Z3 Z6];

Q10=[Z1 Z3 Z7];

Q11=[Z1 Z3 Z8];

Q12=[Z1 Z4 Z5];

Q13=[Z1 Z4 Z6];

Q14=[Z1 Z4 Z7];

Q15=[Z1 Z4 Z8];

Q16=[Z1 Z5 Z6];

Q17=[Z1 Z5 Z7];

Q18=[Z1 Z5 Z8];

Q19=[Z1 Z6 Z7];

Q20=[Z1 Z6 Z8];

Q21=[Z1 Z7 Z8];

Q22=[Z2 Z3 Z4];

Q23=[Z2 Z3 Z5];

Q24=[Z2 Z3 Z6];

Q25=[Z2 Z3 Z7];

Q26=[Z2 Z3 Z8];

Q27=[Z2 Z4 Z5];

Q28=[Z2 Z4 Z6];

Q29=[Z2 Z4 Z7];

Q30=[Z2 Z4 Z8];

Q31=[Z2 Z5 Z6];

Q32=[Z2 Z5 Z7];

Q33=[Z2 Z5 Z8];

Q34=[Z2 Z6 Z7];

Q35=[Z2 Z6 Z8];

Q36=[Z2 Z7 Z8];

Q37=[Z3 Z4 Z5];

Q38=[Z3 Z4 Z6];

Q39=[Z3 Z4 Z7];

Q40=[Z3 Z4 Z8];

Q41=[Z3 Z5 Z6];

Q42=[Z3 Z5 Z7];

Q43=[Z3 Z5 Z8];

Q44=[Z3 Z6 Z7];

Q45=[Z3 Z6 Z8];

Q46=[Z3 Z7 Z8];

Q47=[Z4 Z5 Z6];

Q48=[Z4 Z5 Z7];

Q49=[Z4 Z5 Z8];

Q50=[Z4 Z6 Z7];

Q51=[Z4 Z6 Z8];

Q52=[Z4 Z7 Z8];

Q53=[Z5 Z6 Z7];

Q54=[Z5 Z6 Z8];

Q55=[Z5 Z7 Z8];

Q56=[Z6 Z7 Z8];

%ju01=xlsread('h11.xls');

a=normrnd(0,0.00000000001,q3,1);

ju11=[Q56];

y1=tou(ju11);

[o,r]=size(y1);

for k=1:o

if(y1(k,1)>2)

for i=1:y1(k,1)

for j=1:y1(k,1)

A1(i,j)=cal_dis1(ju11(y1(k,i+1),:),ju11(y1(k,j+1),:));

end

end

B1=sum(A1);

amin=min(B1);

for i=1:y1(k,1)

if(B1(i)==amin)

y1(k,i+1)

ju31(k,:)=ju11(y1(k,i+1),:);

end

end

elseif(y1(k,1)==2)

ju31(k,:)=(ju11(y1(k,2),:)+ju11(y1(k,3),:))/2;

else

display('error')

end

clear A1;

end

[m6,n6]= size(y1);

a11=y1(:,2:n6);

c11= max(a11,[],2);

for i=1:m6

b11(i)=i;

end

e11=[b11' c11];

[i11,j11]=find(e11(:,2)<q6+q4+1);

g11=a11(i11,:);

[m7,n7]= size(g11);

c12= max(g11,[],2);

for i=1:m7

b12(i)=i;

end

e12=[b12' c12];

[i12,j12]=find(e12(:,2)>q6);

g12=g11(i12,:);

[u2,w2]= size(g12);

h2=reshape(g12.',1,u2*w2);

s2=h2';

f2=unique(s2);

[o2,r2]= size(f2);

v12=f2(1:o2,:);

[u1,w1]= size(g11);

h1=reshape(g11.',1,u1*w1);

s1=h1';

f1=unique(s1);

[o1,r1]= size(f1);

v11=f1(1:o1,:);

A freely available MATLAB implemented to perform Icc-cluster

for a data set.

tic

clc

clear all

J1=xlsread('J1.xlsx');

J2=xlsread('J2.xlsx');

J3=xlsread('J3.xlsx');

J4=xlsread('J4.xlsx');

H1=xlsread('H1.xlsx');

J5=[J1 J2];

J6=[J1 J3];

J7=[J1 J4];

J8=[J2 J3];

J9=[J2 J4];

J10=[J3 J4];

J11=[J1 J2 J3];

J12=[J1 J2 J4];

J13=[J1 J3 J4];

J14=[J2 J3 J4];

J15=[J1 J2 J3 J4];

J16=[J1 J2 H1];

J17=[J1 J3 H1];

J18=[J2 J3 H1];

J19=[J2 J4 H1];

J20=[J3 J4 H1];

J21=[J1 J2 J3 H1];

J22=[J1 J2 J4 H1];

J23=[J1 J3 J4 H1];

J24=[J2 J3 J4 H1];

J25=[J1 J2 J3 J4 H1];

J26=[J1 J4 H1];

J27=[J1 H1];

J28=[J2 H1];

J29=[J3 H1];

J30=[J4 H1];

s=1380;

w=J30;

%xlswrite('fpoints.xls',w);

matrix=w;

[m,n]=size(matrix);

bianyi=0.0;

for i=1:m

for j=1:n

matrix(i,j)=matrix(i,j)+bianyi;

end

end

for i=1:m

for j=1:m

dis_matr(i,j)=cal_dis2(matrix(i,:),matrix(j,:));

end

end

[a1,b1]=max(dis_matr);

[a11,a]=max(a1);

b=b1(a);

centroid=[a,b];

for k=1:s

dis_min=zeros(m,1);

num_cen=length(centroid);

for i=1:m

dis_min(i)=dis_matr(i,centroid(1));

for j=1:num_cen

dis_min(i)=min(dis_min(i),dis_matr(i,centroid(j)));

end

end

[dis_max,index]=max(dis_min);

centroid=[centroid,index]; %把找到的质心加到质心集合中

end

%把其余的样本分成24类

belong=zeros(m,1);

for i=1:m

dis_min2=dis_matr(i,centroid(1))*ones(25,1);

for j=1:s

dis_min2(j)=dis_matr(i,centroid(j));

end

[dis_min,index]=min(dis_min2);

belong(i)=index;

end

%循环直到质心不再发生变化

while(1)

centroid2=centroid;

for i=1:s

%计算每一类样本个数

seq=find(belong==i);

num=length(seq);

%寻找新的24个质心

for r=1:num

for t=1:num

dis_matr2(r,t)=cal_dis2(matrix(seq(r),:),matrix(seq(t),:));

end

end

dis_in=zeros(num,1);

for r=1:num

for t=1:num

dis_in(r)=dis_in(r)+dis_matr2(r,t);

end

end

[dis_min,index]=min(dis_in);

centroid(i)=seq(index);

end

if(centroid==centroid2)

break;

end

belong=zeros(m,1);

%把其余的样本分成24类

for i=1:m

dis_min2=dis_matr(i,centroid(1))*ones(25,1);

for j=1:s

dis_min2(j)=dis_matr(i,centroid(j));

end

[dis_min,index]=min(dis_min2);

belong(i)=index;

end

end

%24个类的质心

disp('s个类的质心');

centroid

%24个质心数据

disp('s个质心数据');

for i=1:s

cen(i,:)=matrix(centroid(i),:);

end

cen

%24个类每个类的个数

disp('s个类每个类的个数')

for i=1:s

aa1(i)=length(find(belong==i));

end

f=xlsread('jjs.xls');

a=[belong f];

[b, pos] = sort(a( :,1));

a = a(pos,:);

for i1=1:s

d(i1,:)=sum(a(:,1)==i1);

end

e=cumsum(d);

f=zeros(s,1);

h1=[a(1:e(1,1),3)' f((e(1,1)+1):s,1)'];

for i2=2:s

g(i2-1,:) = [a(e(i2-1,1)+1:(e(i2,1)),3)' f((e(i2,1)-e(i2-1,1)+1):s,1)'];

end

x5=[h1;g];

[t1,t2]=size(x5);

x7=reshape(x5',[],1);;

for i=1:t1

x6(i,1)= sum(x5(i,:)<167)-sum(x5(i,:)<1)-sum(x5(i,:)==5)-sum(x5(i,:)==10)-sum(x5(i,:)==15)-sum(x5(i,:)==20)-sum(x5(i,:)==25)-sum(x5(i,:)==30)-sum(x5(i,:)==35)-sum(x5(i,:)==40)-sum(x5(i,:)==45)-sum(x5(i,:)==50)-sum(x5(i,:)==55)-sum(x5(i,:)==60)-sum(x5(i,:)==65)-sum(x5(i,:)==70)-sum(x5(i,:)==75)-sum(x5(i,:)==80)-sum(x5(i,:)==85)-sum(x5(i,:)==90)-sum(x5(i,:)==95)-sum(x5(i,:)==100)-sum(x5(i,:)==105)-sum(x5(i,:)==110)-sum(x5(i,:)==115)-sum(x5(i,:)==120)-sum(x5(i,:)==125)-sum(x5(i,:)==130)-sum(x5(i,:)==135)-sum(x5(i,:)==140)-sum(x5(i,:)==145)-sum(x5(i,:)==150)-sum(x5(i,:)==155)-sum(x5(i,:)==160)-sum(x5(i,:)==165);

x6(i,2)= sum(x5(i,:)<319)-sum(x5(i,:)<167)-sum(x5(i,:)==170) -sum(x5(i,:)==175) -sum(x5(i,:)==180) -sum(x5(i,:)==185) -sum(x5(i,:)==190) -sum(x5(i,:)==195) -sum(x5(i,:)==200) -sum(x5(i,:)==205) -sum(x5(i,:)==210) -sum(x5(i,:)==215) -sum(x5(i,:)==220) -sum(x5(i,:)==225) -sum(x5(i,:)==230) -sum(x5(i,:)==235) -sum(x5(i,:)==240) -sum(x5(i,:)==245) -sum(x5(i,:)==250) -sum(x5(i,:)==255) -sum(x5(i,:)==260) -sum(x5(i,:)==265) -sum(x5(i,:)==270) -sum(x5(i,:)==275) -sum(x5(i,:)==280) -sum(x5(i,:)==285) -sum(x5(i,:)==290) -sum(x5(i,:)==295) -sum(x5(i,:)==300) -sum(x5(i,:)==305) -sum(x5(i,:)==310) -sum(x5(i,:)==315);

x6(i,3)= sum(x5(i,:)<520)-sum(x5(i,:)<319)-sum(x5(i,:)==320) -sum(x5(i,:)==325) -sum(x5(i,:)==330) -sum(x5(i,:)==335) -sum(x5(i,:)==340) -sum(x5(i,:)==345) -sum(x5(i,:)==350) -sum(x5(i,:)==355) -sum(x5(i,:)==360) -sum(x5(i,:)==365) -sum(x5(i,:)==370) -sum(x5(i,:)==375) -sum(x5(i,:)==380) -sum(x5(i,:)==385) -sum(x5(i,:)==390) -sum(x5(i,:)==395) -sum(x5(i,:)==400) -sum(x5(i,:)==405) -sum(x5(i,:)==410) -sum(x5(i,:)==415) -sum(x5(i,:)==420) -sum(x5(i,:)==425) -sum(x5(i,:)==430) -sum(x5(i,:)==435) -sum(x5(i,:)==440) -sum(x5(i,:)==445) -sum(x5(i,:)==450) -sum(x5(i,:)==455) -sum(x5(i,:)==460) -sum(x5(i,:)==465) -sum(x5(i,:)==470) -sum(x5(i,:)==475) -sum(x5(i,:)==480) -sum(x5(i,:)==485) -sum(x5(i,:)==490) -sum(x5(i,:)==495) -sum(x5(i,:)==500) -sum(x5(i,:)==505) -sum(x5(i,:)==510) -sum(x5(i,:)==515);

x6(i,4)= sum(x5(i,:)<707)-sum(x5(i,:)<520)-sum(x5(i,:)==520) -sum(x5(i,:)==525) -sum(x5(i,:)==530) -sum(x5(i,:)==535) -sum(x5(i,:)==540) -sum(x5(i,:)==545) -sum(x5(i,:)==550) -sum(x5(i,:)==555) -sum(x5(i,:)==560) -sum(x5(i,:)==565) -sum(x5(i,:)==570) -sum(x5(i,:)==575) -sum(x5(i,:)==580) -sum(x5(i,:)==585) -sum(x5(i,:)==590) -sum(x5(i,:)==595) -sum(x5(i,:)==600) -sum(x5(i,:)==605) -sum(x5(i,:)==610) -sum(x5(i,:)==615) -sum(x5(i,:)==620) -sum(x5(i,:)==625) -sum(x5(i,:)==630) -sum(x5(i,:)==635) -sum(x5(i,:)==640) -sum(x5(i,:)==645) -sum(x5(i,:)==650) -sum(x5(i,:)==655) -sum(x5(i,:)==660) -sum(x5(i,:)==665) -sum(x5(i,:)==670) -sum(x5(i,:)==675) -sum(x5(i,:)==680) -sum(x5(i,:)==685) -sum(x5(i,:)==690) -sum(x5(i,:)==695) -sum(x5(i,:)==700) -sum(x5(i,:)==705);

x6(i,5)= sum(x5(i,:)<859)-sum(x5(i,:)<707)-sum(x5(i,:)==710) -sum(x5(i,:)==715) -sum(x5(i,:)==720) -sum(x5(i,:)==725) -sum(x5(i,:)==730) -sum(x5(i,:)==735) -sum(x5(i,:)==740) -sum(x5(i,:)==745) -sum(x5(i,:)==750) -sum(x5(i,:)==755) -sum(x5(i,:)==760) -sum(x5(i,:)==765) -sum(x5(i,:)==770) -sum(x5(i,:)==775) -sum(x5(i,:)==780) -sum(x5(i,:)==785) -sum(x5(i,:)==790) -sum(x5(i,:)==795) -sum(x5(i,:)==800) -sum(x5(i,:)==805) -sum(x5(i,:)==810) -sum(x5(i,:)==815) -sum(x5(i,:)==820) -sum(x5(i,:)==825) -sum(x5(i,:)==830) -sum(x5(i,:)==835) -sum(x5(i,:)==840) -sum(x5(i,:)==845) -sum(x5(i,:)==850) -sum(x5(i,:)==855);

x6(i,6)= sum(x5(i,:)<963)-sum(x5(i,:)<859)-sum(x5(i,:)==860) -sum(x5(i,:)==865) -sum(x5(i,:)==870) -sum(x5(i,:)==875) -sum(x5(i,:)==880) -sum(x5(i,:)==885) -sum(x5(i,:)==890) -sum(x5(i,:)==895) -sum(x5(i,:)==900) -sum(x5(i,:)==905) -sum(x5(i,:)==910) -sum(x5(i,:)==915) -sum(x5(i,:)==920) -sum(x5(i,:)==925) -sum(x5(i,:)==930) -sum(x5(i,:)==935) -sum(x5(i,:)==940) -sum(x5(i,:)==945) -sum(x5(i,:)==950) -sum(x5(i,:)==955) -sum(x5(i,:)==960);

x6(i,7)= sum(x5(i,:)<1092)-sum(x5(i,:)<963)-sum(x5(i,:)==965) -sum(x5(i,:)==970) -sum(x5(i,:)==975) -sum(x5(i,:)==980) -sum(x5(i,:)==985) -sum(x5(i,:)==990) -sum(x5(i,:)==995) -sum(x5(i,:)==1000) -sum(x5(i,:)==1005) -sum(x5(i,:)==1010) -sum(x5(i,:)==1015) -sum(x5(i,:)==1020) -sum(x5(i,:)==1025) -sum(x5(i,:)==1030) -sum(x5(i,:)==1035) -sum(x5(i,:)==1040) -sum(x5(i,:)==1045) -sum(x5(i,:)==1050) -sum(x5(i,:)==1055) -sum(x5(i,:)==1060) -sum(x5(i,:)==1065) -sum(x5(i,:)==1070) -sum(x5(i,:)==1075) -sum(x5(i,:)==1080) -sum(x5(i,:)==1085) -sum(x5(i,:)==1090) -sum(x5(i,:)==1095) -sum(x5(i,:)==1100) -sum(x5(i,:)==1105);

x6(i,8)= sum(x5(i,:)<1206)-sum(x5(i,:)<1092) -sum(x5(i,:)==1110) -sum(x5(i,:)==1115) -sum(x5(i,:)==1120) -sum(x5(i,:)==1125) -sum(x5(i,:)==1130) -sum(x5(i,:)==1135) -sum(x5(i,:)==1140) -sum(x5(i,:)==1145) -sum(x5(i,:)==1150) -sum(x5(i,:)==1155) -sum(x5(i,:)==1160) -sum(x5(i,:)==1165) -sum(x5(i,:)==1170) -sum(x5(i,:)==1175) -sum(x5(i,:)==1180) -sum(x5(i,:)==1185) -sum(x5(i,:)==1190) -sum(x5(i,:)==1195) -sum(x5(i,:)==1200) -sum(x5(i,:)==1205);

x6(i,9)= sum(x5(i,:)<1320)-sum(x5(i,:)<1206)-sum(x5(i,:)==1210) -sum(x5(i,:)==1215) -sum(x5(i,:)==1220) -sum(x5(i,:)==1225) -sum(x5(i,:)==1230) -sum(x5(i,:)==1235) -sum(x5(i,:)==1240) -sum(x5(i,:)==1245) -sum(x5(i,:)==1250) -sum(x5(i,:)==1255) -sum(x5(i,:)==1260) -sum(x5(i,:)==1265) -sum(x5(i,:)==1270) -sum(x5(i,:)==1275) -sum(x5(i,:)==1280) -sum(x5(i,:)==1285) -sum(x5(i,:)==1290) -sum(x5(i,:)==1295) -sum(x5(i,:)==1300) -sum(x5(i,:)==1305) -sum(x5(i,:)==1310) -sum(x5(i,:)==1315);

x6(i,10)= sum(x5(i,:)<1524)-sum(x5(i,:)<1320)-sum(x5(i,:)==1320) -sum(x5(i,:)==1325) -sum(x5(i,:)==1330) -sum(x5(i,:)==1335) -sum(x5(i,:)==1340) -sum(x5(i,:)==1345) -sum(x5(i,:)==1350) -sum(x5(i,:)==1355) -sum(x5(i,:)==1360) -sum(x5(i,:)==1365) -sum(x5(i,:)==1370) -sum(x5(i,:)==1375) -sum(x5(i,:)==1380) -sum(x5(i,:)==1385) -sum(x5(i,:)==1390) -sum(x5(i,:)==1395) -sum(x5(i,:)==1400) -sum(x5(i,:)==1405) -sum(x5(i,:)==1410) -sum(x5(i,:)==1415) -sum(x5(i,:)==1420) -sum(x5(i,:)==1425) -sum(x5(i,:)==1430) -sum(x5(i,:)==1435) -sum(x5(i,:)==1440) -sum(x5(i,:)==1445) -sum(x5(i,:)==1450) -sum(x5(i,:)==1455) -sum(x5(i,:)==1460) -sum(x5(i,:)==1465) -sum(x5(i,:)==1470) -sum(x5(i,:)==1475) -sum(x5(i,:)==1480) -sum(x5(i,:)==1485) -sum(x5(i,:)==1490) -sum(x5(i,:)==1495) -sum(x5(i,:)==1500) -sum(x5(i,:)==1505) -sum(x5(i,:)==1510) -sum(x5(i,:)==1515) -sum(x5(i,:)==1520);

x6(i,11)= sum(x5(i,:)<1726)-sum(x5(i,:)<1524)-sum(x5(i,:)==1525) -sum(x5(i,:)==1530) -sum(x5(i,:)==1535) -sum(x5(i,:)==1540) -sum(x5(i,:)==1545) -sum(x5(i,:)==1550) -sum(x5(i,:)==1555) -sum(x5(i,:)==1560) -sum(x5(i,:)==1565) -sum(x5(i,:)==1570) -sum(x5(i,:)==1575) -sum(x5(i,:)==1580) -sum(x5(i,:)==1585) -sum(x5(i,:)==1590) -sum(x5(i,:)==1595) -sum(x5(i,:)==1600) -sum(x5(i,:)==1605) -sum(x5(i,:)==1610) -sum(x5(i,:)==1615) -sum(x5(i,:)==1620) -sum(x5(i,:)==1625) -sum(x5(i,:)==1630) -sum(x5(i,:)==1635) -sum(x5(i,:)==1640) -sum(x5(i,:)==1645) -sum(x5(i,:)==1650) -sum(x5(i,:)==1655) -sum(x5(i,:)==1660) -sum(x5(i,:)==1665) -sum(x5(i,:)==1670) -sum(x5(i,:)==1675) -sum(x5(i,:)==1680) -sum(x5(i,:)==1685) -sum(x5(i,:)==1690) -sum(x5(i,:)==1695) -sum(x5(i,:)==1700) -sum(x5(i,:)==1705) -sum(x5(i,:)==1710) -sum(x5(i,:)==1715) -sum(x5(i,:)==1720) -sum(x5(i,:)==1725);

end

for i = 1:t1

x2(i,:)=x6(i,:)/(sum(x6(i,:)));

end

for i=1:t1

Z((t2)*(i-1)+1:(t2)*i,:)=repmat(x2(i,:),t2,1);

end

B=[x7 Z];

B(B(:,1)==0, :) = [];

[~,idx]=sort(B(:,1));

C=B(idx,:);

C(find(isnan(C)==1)) = 0;

xlswrite('C1.xls',C)
